# Assessment of Intravenous Fluid Prescribing and Cannulation Appropriateness in an Acute Medical Unit: A Prospective Service Evaluation

**DOI:** 10.7759/cureus.109364

**Published:** 2026-05-21

**Authors:** Amr Barghout, Tim Harris, Kristie Kear, Megan Cocker, Prashant Parulekar

**Affiliations:** 1 Acute Medicine and General Medicine, East Kent Hospitals NHS Trust, Kent, GBR; 2 Emergency Medicine, Queen Mary University of London, London, GBR; 3 General Internal Medicine, University Hospitals Sussex NHS Foundation Trust, Brighton and Hove, GBR

**Keywords:** fluid balance, intravenous cannulation, intravenous fluids, patient’s safety, quality improvement (qi), service evaluation and improvement

## Abstract

Background

Intravenous (IV) fluid therapy and peripheral venous cannulation (PVC) are central to acute medical care, yet inappropriate IV fluid prescribing and unnecessary cannulation can expose patients to avoidable risks. Acute Medical Units (AMUs) are high‑turnover environments where IV fluids and cannulas are often initiated early, but may not be routinely reviewed once patients stabilise and can drink orally.

Objective

This study aimed to evaluate the appropriateness of IV fluid prescribing and peripheral cannulation in an AMU, particularly in patients able to maintain oral hydration, and to assess the quality of fluid balance documentation.

Methods

A prospective service evaluation was conducted in the AMU of a district general hospital between March and April 2025. Adult patients (≥16 years) admitted during the study period who were clinically able to swallow were eligible. Data were collected for 200 consecutive admissions following a 20‑patient pilot. Information was obtained from bedside review, fluid balance charts, and electronic records. Variables included IV fluid use, oral intake status, presence and indication for peripheral cannulation, prescriber seniority, and completeness of input-output documentation. Data were analysed descriptively using simple counts, percentages, and summary statistics.

Results

Of 200 patients, the median age was 76 years (range 18-96), and 89% had a peripheral cannula in situ. Overall, 18% received IV fluids during their AMU stay. Among those receiving IV fluids only, all were able to take oral fluids, and none had fully completed input-output charts. The majority of IV fluids were prescribed by senior clinicians. Fluid balance documentation was limited: only a minority of patients had fluid charts in place, and of these, just over a quarter were completed accurately. Among cannulated patients, almost one‑third had no clear documented clinical indication for IV access.

Conclusions

In this AMU, IV fluids were frequently administered to patients who were able to maintain oral intake; fluid balance documentation was often incomplete, and a substantial proportion of peripheral cannulas lacked a clear indication. These findings highlight opportunities to improve IV fluid stewardship, strengthen documentation, and review cannulation practice to reduce avoidable risk and support safer, more efficient acute medical care.

## Introduction

Intravenous (IV) fluid therapy is a cornerstone of acute medical management; however, inappropriate prescribing and inadequate monitoring remain frequent challenges in hospital practice. The National Institute for Health and Care Excellence guidance emphasises that fluid assessment should be undertaken daily and that IV fluids should only be used when patients’ needs cannot be met orally or enterally, with prescribing ideally undertaken by experienced clinicians [1]. Despite these clear recommendations, approximately one in five patients receiving IV fluids experience complications due to inappropriate administration [1].

Recent evidence highlights persistent gaps between recommended practice and real‑world fluid management. A 2023 UK audit reported that only 10% of patients received regular fluid status assessments, 32% were given correct resuscitation fluids, 52% received appropriately indicated maintenance fluids, and critically, none received maintenance fluids with correct electrolyte composition [2]. These findings reinforce the need for robust stewardship and consistent adherence to established standards in fluid prescribing.

IV therapy is also associated with notable device‑related risks. Peripheral venous cannulas (PVCs) account for 10-20% of healthcare‑associated infections (HCAIs) in the United Kingdom, with up to 70% of these infections estimated to be preventable [3]. HCAIs cost the NHS approximately £2.7 billion per year, suggesting cannula‑related infections may contribute between £270 million and £540 million annually [3]. Beyond infection, PVC‑associated thrombophlebitis is another prevalent complication, with incidence rates ranging widely from 2% to 80% [4].

Despite the clinical and financial implications, IV fluid use and cannulation practices within Acute Medical Units (AMUs) remain underexplored. The AMU environment is typically characterised by high patient turnover and rapid clinical decision‑making, where IV fluids and cannulas are often initiated early and may not always be routinely reviewed once patients become clinically stable or capable of oral hydration. Understanding current practices in AMU settings is therefore an important step towards improving safety, reducing avoidable complications, and supporting alignment with national best‑practice recommendations.

Although the appropriateness of IV fluids for patients able to maintain oral hydration has been well-described in national guidance, our objective was to provide a local, AMU‑specific description of how IV fluid use and peripheral cannulation occur together in routine practice to inform quality improvement. The aim of this study was to evaluate the appropriateness of IV fluid prescribing and peripheral cannulation within the AMU, specifically assessing whether IV fluids are being administered to patients who are clinically capable of maintaining adequate hydration orally, and to identify the extent of unnecessary cannulation in this acute care setting.

## Materials and methods

This prospective service evaluation was conducted within the AMU at William Harvey Hospital and aimed to assess the appropriateness of IV fluid prescribing and peripheral cannulation practices among adult medical admissions. Data were collected over a two‑month period between March and April 2025, during which the AMU experienced its usual high turnover of acute medical presentations. The project was registered with the East Kent Hospitals University NHS Foundation Trust Research and Innovation Department (reference: 2025/GAP/18) as a service evaluation and, in accordance with institutional policy, did not require formal Research Ethics Committee review or individual patient consent.

Patients were eligible for inclusion if they were 16 years or older, admitted to the AMU during the study period, and clinically able to swallow and tolerate oral fluids. The ability to eat and drink orally was defined as the clinical capacity to swallow and tolerate oral fluids, rather than quantified oral intake volumes. Patients were excluded if they required IV access or fluids for specific clinical reasons such as acute instability or seizure management. Patients arriving directly from the emergency department (ED) to the AMU without sufficient time for assessment were also excluded, as the ED and AMU function as separate but sequential stages of acute care in our hospital.

Following an initial pilot involving 20 patients, full data collection was undertaken for 200 consecutive AMU admissions. Data were gathered by the study team, with logistical support from the AMU nursing staff in identifying eligible patients and locating fluid balance charts. Information was extracted from bedside assessments, electronic medical records, and fluid balance charts. Variables recorded included patient demographics (age, sex, length of stay), IV fluid prescription details (type of fluid, indication, grade of prescriber (consultant, registrar, senior house officer)), oral intake status, and the presence and accuracy of input-output monitoring. Additional data on peripheral cannulation were collected, including whether a cannula was present, its indication, and whether its insertion was deemed appropriate based on documented clinical need.

The primary outcomes were as follows: (1) the proportion of patients receiving IV fluids despite being clinically able to take oral fluids and (2) the use and completeness of fluid balance documentation. Secondary outcomes included the rate of inappropriate peripheral cannulation, the types of IV fluids administered, and the seniority of the clinicians making IV fluid decisions.

Findings were presented using summary statistics to characterise current practice patterns. Data were analysed descriptively, with frequencies and percentages used for categorical variables and medians with ranges and means used for continuous variables; no inferential statistical testing was performed.

## Results

A total of 200 patients were included in the evaluation (Table 1). The median age was 76 years (range: 18-96), with a mean age of 71.6 years. The cohort comprised 92 women (46%) and 108 men (54%). Length of stay in the AMU ranged from zero hours to 30 days, reflecting the typical variability of patient turnover within this setting.

**Table 1:** Patient demographics and baseline characteristics (n = 200) Data presented as n (%) for categorical variables, median (range), and mean for continuous variables. Descriptive statistics only; no inferential statistical testing performed

Characteristic	Value
Total patients (n)	200
Median age, years (range)	76 (18-96)
Mean age, years	71.6
Female, n (%)	92 (46.0)
Male, n (%)	108 (54.0)
Length of stay, hours (range)	0-720 (0 hours-30 days)

IV fluid prescribing

Table 2 presents IV fluid prescribing practices. Among all admissions, 36 patients (18%) received IV fluids at any time during their AMU stay. Of these, 28 patients (13.8%) received IV fluids without other IV medications; a small number of patients also received blood products during their stay. Notably, all patients in this IV‑fluids‑only subgroup were clinically able to eat and drink orally, although oral intake volumes were not systematically quantified. Despite receiving IV therapy, none of these patients had contemporaneous input‑output charting in place.

**Table 2:** IV prescribing practices IV: intravenous fluid; AMU: Acute Medical Unit Data presented as n (%). "IV fluids only" excludes patients receiving other IV medications; patients who also received blood products remain included in the overall IV fluid cohort

Variable	n	Percentage (%)
Patients receiving IV fluids during AMU stay	36	18.0
Patients receiving IV fluids only*	28	13.8
Able to eat and drink orally (of the IV fluids-only group)	28	100.0
Completed input-output charts (of IV fluids-only group)	0	0.0
Prescriber seniority (n = 36):		
Consultant	23	63.0
Registrar	2	5.6
FY2	9	25.0
FY1	1	2.7
Type of IV fluid prescribed (n = 36):		
Plasmalyte	20	55.6
Sodium chloride	4	11.1
Glucose	2	5.6
Potassium-containing fluids	1	2.8
Blood products	2	5.6
Combination regimens	7	19.4

The majority of IV fluid prescriptions were made by senior clinicians, with consultants responsible for 23 prescriptions (63%), followed by Foundation Year 2 (FY2) doctors (9 prescriptions, 25%), registrars (2 prescriptions, 5.6%), and Foundation Year 1 (FY1) doctors (1 prescription, 2.7%). The most frequently prescribed fluid was Plasmalyte in 20 patients (55.6%), followed by sodium chloride in four patients (11.1%), combination regimens in seven patients (19.4%), glucose in two patients (5.6%), blood products in two patients (5.6%), and potassium-containing fluids in one patient (2.8%).

Oral intake and fluid balance documentation

Oral intake status and fluid balance documentation are shown in Table 3. The majority of patients (174 patients, 87%) were able to take fluids orally without assistance. Only three patients (1.5%) were recorded as nil by mouth, and 23 patients (11.5%) required prompting to maintain adequate oral intake.

**Table 3:** Oral intake status and fluid balance documentation Data presented as n (%). Descriptive statistics only; no inferential statistical testing performed

Variable	n	Percentage (%)
Oral intake status (n = 200):		
Nil by mouth	3	1.5
Required prompting for oral intake	23	11.5
Adequate oral intake	174	87.0
Fluid balance documentation (n = 200):		
Input-output charts in place	31	15.5
Charts completed accurately (of those with charts, n = 31)	8	25.8
Charts incomplete or inaccurate (of those with charts, n = 31)	23	74.2

Fluid balance documentation was notably deficient across the cohort. Only 31 patients (15.5%) had input-output charts in place. Of these 31 charts, just eight (25.8%) were completed accurately, while 23 (74.2%) were incomplete or inaccurate.

Peripheral cannulation practices

Table 4 summarises cannulation practices. Peripheral cannulation was highly prevalent, with 178 patients (89%) having a cannula in situ. Among those cannulated, the documented indications varied: 92 patients (51.7%) received IV medications only, 30 patients (16.9%) received both IV fluids and medications, five patients (2.8%) received IV fluids alone, and one patient (0.6%) received only blood products.

**Table 4:** Peripheral cannulation practices IV: intravenous fluid Data presented as n (%). Descriptive statistics only; no inferential statistical testing performed

Variable	n	Percentage (%)
Patients with a peripheral cannula in situ	178	89.0
Indication for cannulation (n = 178):		
IV medications only	92	51.7
IV fluids only	5	2.8
Both IV fluids and medications	30	16.9
Blood products only	1	0.6
Appropriateness of cannulation (n = 178):		
Inappropriately cannulated (no clear indication)	53	29.8
Appropriately cannulated	125	70.2

Overall, around one‑third of cannulated patients were judged to be inappropriately cannulated, with no clear documented clinical indication for IV access, while the remaining majority had appropriate documented indications (Table 4). Cannulation was judged appropriate when there was a clearly documented clinical indication, such as IV medications, IV fluids for resuscitation or replacement, blood products, or anticipated urgent IV therapy, and inappropriate when no such indication was documented in the notes.

## Discussion

This service evaluation identified significant opportunities to improve IV fluid prescribing and peripheral cannulation practices within the AMU. Appropriateness of IV fluid prescribing and cannulation was assessed using predefined criteria, with treatments considered appropriate when a clear documented indication (e.g. IV medications, fluid resuscitation or replacement, or blood products) was present, and inappropriate when no such indication was documented. A key finding was that IV fluids were frequently administered to patients who were clinically able to maintain hydration orally. However, we recognise that in an AMU setting, PVC is sometimes undertaken prophylactically to secure rapid IV access in patients with unstable or evolving clinical trajectories, and this rationale may not always be fully reflected in the documentation. This mirrors wider concerns that inappropriate IV fluid therapy remains common, despite clear guidance that IV fluids should be used only when oral or enteral intake cannot meet patient needs [1]. Similar discrepancies between recommended and actual practice have been reported internationally [5], with incorrect fluid types, inappropriate volumes, and the omission of key clinical assessments (such as weight and electrolytes) frequently cited as causes of misprescribing in hospitalised adults.

The lack of adequate fluid balance documentation in the AMU, where only a minority of patients had input-output charts completed correctly, is consistent with broader evidence demonstrating deficiencies in hydration monitoring [6]. Previous work has noted that junior doctors often lack training in safe fluid prescribing and that poor documentation contributes to fluid overload, electrolyte imbalance, and avoidable harm. Furthermore, a recent UK audit found that none of the patients receiving maintenance fluids was prescribed solutions with an appropriate electrolyte composition [2], reinforcing a national pattern of inconsistent adherence to established prescribing standards.

Cannulation practices within the AMU also require attention. Nearly one‑third of patients in this evaluation had no clear indication for cannulation, raising concerns about unnecessary invasive procedures. This finding aligns with evidence showing high rates of PVC complications. Although the overall incidence of PVC‑related bloodstream infection is relatively low, associated complications, including severe sepsis and metastatic infection, carry substantial morbidity and significantly increase hospital costs [7]. International data highlight the scale of the problem, with reports that a large proportion of inpatients receive peripheral catheters and that poor insertion and maintenance practices substantially increase the risk of bloodstream infections and sepsis [8].

The wider literature supports the need for improved training and standardisation of cannulation practice. A multinational consensus review found PVC failure rates of 35-50% and highlighted gaps in clinician knowledge and competency [9], even in high‑income settings. Systematic reviews also indicate that preventive strategies such as strict asepsis, appropriate glove use, scheduled catheter replacement, and consistent documentation can reduce the risk of phlebitis and catheter‑related infections [10].

This evaluation has several limitations that warrant consideration. First, the single-centre design and relatively short data collection period may limit generalizability to other AMU settings with different patient populations or staffing models. Second, the 24-hour data capture window may not have captured the full clinical trajectory of patients who subsequently deteriorated or had delayed documentation, and some patients were newly transferred from the ED and had not yet undergone full assessment. Third, assessment of cannulation appropriateness was based on documented indications; some cannulas judged "inappropriate" may have had undocumented but valid clinical rationale, such as anticipated deterioration in seizure patients or patients admitted for conditions where IV access may reasonably be maintained as a precaution. In addition, we did not systematically collect detailed physiological or biochemical markers of volume status or precise oral intake volumes, so IV fluid use in individual patients cannot be definitively classified as inappropriate. Fourth, by design, we excluded patients with clear indications for IV fluids or IV access (e.g. acute instability or seizure management), so our denominator does not represent all AMU admissions and may overestimate the proportion of apparently inappropriate IV fluid use or cannulation within the remaining cohort. Fifth, we did not assess patient outcomes such as complications, length of hospital stay, or readmission rates, which would provide additional evidence of the clinical impact of inappropriate IV fluid use or cannulation. Finally, as a service evaluation, this study describes practice patterns but does not test interventions or establish causality.

Taken together, the findings of this evaluation, supported by the wider literature, suggest that IV fluid therapy and cannulation are often initiated early during acute admission but not consistently reassessed as clinical status improves. Strengthening adherence to evidence‑based guidelines, improving documentation, and enhancing staff training could substantially reduce avoidable complications. These results are consistent with emerging calls for the development of “IV fluid stewardship” frameworks [11], which aim to reduce inappropriate IV fluid use through targeted education, decision‑support tools, and regular multidisciplinary review.

## Conclusions

This service evaluation highlights important opportunities to optimise intravenous fluid therapy and peripheral cannulation practices within the AMU. IV fluids were frequently administered to patients who were clinically able to maintain hydration orally, indicating a gap between guideline‑recommended practice and real‑world decision‑making. Fluid balance documentation was often incomplete, limiting clinicians’ ability to accurately assess hydration status. In addition, a substantial proportion of peripheral cannulas were inserted without a clear documented clinical indication, exposing patients to potentially avoidable invasive procedures. These findings underscore the need for targeted quality improvement initiatives to promote safer and more appropriate use of IV fluids and peripheral cannulas in acute medical care, while future work should evaluate the impact of such initiatives on patient outcomes and resource use.

## References

[1] (2026). NICE. Intravenous fluid therapy in adults in hospital. https://www.nice.org.uk/guidance/cg174/chapter/Recommendations.

[2] Hassan SWU, Alam SN, Shoaib M, Ibrahim M (2023). Preventing avoidable harm with safe IV fluid therapy-a closed loop audit. Br J Surg.

[3] Guest JF, Keating T, Gould D, Wigglesworth N (2020). Modelling the annual NHS costs and outcomes attributable to healthcare-associated infections in England. BMJ Open.

[4] Zhang L, Cao S, Marsh N, Ray-Barruel G, Flynn J, Larsen E, Rickard CM (2016). Infection risks associated with peripheral vascular catheters. J Infect Prev.

[5] Gao X, Huang KP, Wu HY, Sun PP, Yan JJ, Chen J, Chen X (2015). Inappropriate prescribing of intravenous fluid in adult inpatients-a literature review of current practice and research. J Clin Pharm Ther.

[6] Frost P (2015). Intravenous fluid therapy in adult inpatients. BMJ.

[7] Eggers M, Suchomel M (2023). In-vivo efficacy of alcohol-based hand rubs against noroviruses: a novel standardized European test method simulating practical conditions. J Hosp Infect.

[8] (2026). World Health Organization. New guidance aims to reduce bloodstream infections from catheter use. https://www.who.int/news/item/09-05-2024-new-guidance-aims-to-reduce-bloodstream-infections-from-catheter-use.

[9] Thompson J, Steinheiser MM, Hotchkiss JB (2024). Standards of care for peripheral intravenous catheters: evidence-based expert consensus. Br J Nurs.

[10] Dobrescu A, Constantin AM, Pinte L (2024). Effectiveness and safety of measures to prevent infections and other complications associated with peripheral intravenous catheters: a systematic review and meta-analysis. Clin Infect Dis.

[11] Carr JR, Hawkins WA, Newsome AS, Smith SE, Amber B C, Bland CM, Branan TN (2022). Fluid stewardship of maintenance intravenous fluids. J Pharm Pract.

